# Treatment of Polycystic Liver Disease: Impact on Patient-reported Symptom Severity and Health-related Quality of Life

**DOI:** 10.1097/MCG.0000000000001749

**Published:** 2022-08-19

**Authors:** Renée Duijzer, Thijs R.M. Barten, Christian B. Staring, Joost P.H. Drenth, Tom J.G. Gevers

**Affiliations:** * Department of Gastroenterology and Hepatology, Radboud University Medical Center, Nijmegen, the Netherlands; † European Reference Network RARE-LIVER, Hamburg, Germany; ‡ Department of Gastroenterology and Hepatology, Maastricht University Medical Center, Maastricht, the Netherlands

**Keywords:** polycystic liver disease, polycystic kidney disease, autosomal dominant polycystic liver disease, autosomal polycystic kidney disease, treatment, patient-reported outcome measure, symptom severity, quality of life, health-related quality of life, questionnaire

## Abstract

Polycystic liver disease (PLD) is a genetic disorder in which patients suffer from progressive development of multiple (>10) hepatic cysts. Most patients remain asymptomatic during the course of their disease. However, a minority of PLD patients suffer from symptoms caused by hepatomegaly leading to serious limitations in daily life. Untreated symptomatic PLD patients score significantly worse on health-related quality of life (HRQoL) compared to age and gender-matched populations. Currently, liver transplantation is the only curative treatment for PLD. The main goal of other available therapies is to strive for symptomatic relief and improvement of HRQoL by suppressing disease progression. In this review, we summarize the effect of PLD treatment on patient-reported outcome measures with a distinction between HRQoL and symptom severity. At present there is heterogeneity in application of questionnaires and no questionnaire is available that measures both HRQoL and PLD symptom severity. Therefore, we recommend the combination of a validated PLD-specific symptom severity questionnaire and a general HRQoL questionnaire to evaluate treatment success as a minimal core set. However, the specific choice of questionnaires depends on treatment choice and/or research question. These questionnaires may serve as a biomarker of treatment response, failure, and adverse events.

Polycystic liver disease (PLD) is a genetic disorder in which patients suffer from progressive development of multiple (>10) hepatic cysts. The genetic origin of PLD can be divided in 2 etiologies: autosomal dominant polycystic kidney disease (ADPKD) with presence of multiple cysts in kidneys and liver, and autosomal dominant polycystic liver disease (ADPLD) where cysts are confined to the liver. Patients affected by PLD are predominantly female (>80%)^1^ and can be categorized according to severity of liver phenotype. Although symptom burden is the main indication for treatment, the liver phenotype can aid in determining treatment choice.^2^

The natural disease course of PLD remains unknown. Although cyst burden may increase, liver function is generally preserved and most patients remain asymptomatic during the course of the disease. A minority of PLD patients suffer from symptoms caused by hepatomegaly or strategically located cysts. Although this group is small, these symptoms can lead to serious limitations in daily life when patients experience a high symptom burden. Commonly reported symptoms include abdominal distention, pain, early satiety, weight loss, nausea, vomiting, dyspnea, and restriction in range of motion. In contrast, specific biliary symptoms such as jaundice (3%) or cholangitis (1%) are rare.^3^–^5^ Portal hypertension and occurrence of esophageal varices are extremely rare in PLD, and may occur owing to strategically located cysts.

PLD-specific symptom patient-reported outcome measures (PROMs) have been developed which differentiate between PLD patients and those from the general population depending on PLD-related symptoms.^6^ Moreover, untreated symptomatic PLD patients score significantly worse on health-related quality of life (HRQoL) measures such as EuroQol-visual analog scales (EQ-VAS) and Short Form 36 (SF-36) Physical Component Summary (PCS) score compared with age and gender-matched populations.^3^,^6^,^7^ Most studies using the SF-36 do not find a lowered mental component summary (MCS) score compared with the reference population, however one study assessed the MCS using the Short Form 12 (SF-12) and did detect a significant difference.^6^

Currently, liver transplantation is the only curative treatment option for PLD. The main goal of other available therapies is to achieve symptomatic relief and improvement of HRQoL by suppressing progression of disease. However, these therapies all possess an unique efficacy/safety balance that may come with technical success yet compromise HRQoL. Many studies have taken reduction of (height-adjusted) total liver volume ((h)TLV) or total cyst volume (TCV) as the primary outcome measures to determine treatment success. These surrogate endpoints might be less meaningful to patients compared with symptom burden or HRQoL depending on the type of treatment. For example, a previous study demonstrated that a larger hTLV correlated with lower SF-36 scores and a higher symptom severity.^3^ This correlation was yet not observed after aspiration sclerotherapy, where symptom relief determined the patient-reported rating of change and not volume reduction in patients with dominant hepatic cysts.^8^

This raises the question whether liver or cyst volume alone is the optimal outcome measure to determine treatment efficacy or that this should be combined with PROMs that evaluate treatment impact using the patient’s HRQoL and symptom severity. In this article we will review the current literature on HRQoL in PLD, with special emphasis on the effects of currently available therapies on these PROMs.

## METHODS

PubMed was searched for terms related to PLD, HRQoL, and the different treatments discussed in this article, on February 28, 2022. Additional (original) articles were identified through literature snowballing. Studies were included when they were written in English and addressed PLD treatment in combination with either HRQoL or symptom severity. Supplementary file 1, Supplemental Digital Content 1, http://links.lww.com/JCG/A886 contains an overview of the search strategy.

## QUESTIONNAIRES

Many factors can affect a patient’s HRQoL, including disease-specific symptoms. The most objective measures currently available to measure (factors of) self-rated HRQoL are PROMs. In total 16 articles were identified, yielding 10 different questionnaires which were queried at more than 10 different time moments. These findings are summarized in Table 1.

**TABLE 1:** Overview of Included Studies

Title	Year	Country	Type of Study	N patients	Disease	Intervention	Intervention Duration	PROMs Used
Medical								
Keimpema et al^9^	2009	Netherlands and Belgium	Randomized controlled trial	54	ADPKD and ADPLD	Lanreotide 120 mg/4 wk	6 mo	SF-36 GI questionnaire
Hogan et al^10^	2010	USA	Randomized controlled trial	42	ADPKD and ADPLD	Octreotide 40 mg/4 wk	12 mo	SF-36
Gevers et al^11^	2015	Netherlands	Prospective cohort	43	ADPKD	Lanreotide 120 mg/4 wk	6 mo	EQ-5D GI questionnaire
Aerts et al^12^	2019	Netherlands	Randomized controlled trial	175	ADPKD	Lanreotide 120 mg/4 wk	120 weeks	GI questionnaire
Hogan et al^13^	2020	USA	Randomized controlled trial	48	ADPKD and ADPLD	Pasireotide 60 mg/4 wk	12 mo	SF-36 GI questionnaire
Chrispijn et al^14^	2013	Netherlands	Randomized controlled trial	44	ADPKD and ADPLD	Octreotide 40 mg/4 wk and everolimus 2.5 mg/daily	12 mo	EQ-5D GI questionnaire
Anderegg et al^15^	2020	Switzerland	Prospective cohort	38	ADPKD and ADPLD	Tolvaptan 90/30 mg/daily	12 mo	KDQOL-SF
D’angolo et al^16^	2016	Spain and Netherlands	Randomized controlled trial	34	ADPLD	Ursochol 15–20 mg/kg/day	24 weeks	SF-36 EQ-VAS EORTC-QLQ-c30 PLD-Q
Radiologic								
Benzimra et al^17^	2014	France	Retrospective cohort	57	Symptomatic hepatic cysts	AS	N/A	Self-designed questionnaire
Neijenhuis et al^8^	2019	Netherlands	Randomized controlled trial	32	Symptomatic hepatic cysts	AS	N/A	PLD-Q
Surgical								
Schnelldorfer et al^5^	2009	USA	Retrospective cohort	141	ADPKD and ADPLD	Mixed (CF/resection/LT)	N/A	SF-36 Symptom status ECOG-PS
Ogawa et al^18^	2014	Japan	Retrospective cohort	202	ADPLD	Mixed (AS/CF/resection/LT)	N/A	ECOG-PS
Keimpema et al^19^	2008	Netherlands	Prospective cohort	12	ADPLD	CF	N/A	GI questionnaire
Bernts et al^6^	2020	USA	Prospective cohort	18	ADPLD	PHCF	N/A	SF-12 EQ-VAS PLD-Q
Boillot et al^20^	2021	France	Retrospective cohort	29	ADPLD	PHCF	N/A	ECOG-PS
Kirchner et al^21^	2006	Germany	Retrospective cohort	36	ADPKD and ADPLD	CKLT	N/A	SF-36 Self-designed questionnaire
Ding et al^22^	2019	China	Retrospective cohort	11	ADPLD	LT	N/A	SF-36 ECOG-PS

ADPKD indicates autosomal dominant polycystic kidney disease; ADPLD, autosomal dominant polycystic liver disease; AS, aspiration sclerotherapy; CF, cyst fenestration; CKLT, combined kidney-liver transplantation; ECOG-PS, Eastern Cooperative Oncology Group Performance Status; EORTC-QLQ-c30, European Organization for the Research and Treatment of Cancer Quality of Life Questionnaire; EQ-5D, EuroQol 5 Dimensions; EQ-VAS, EuroQol-visual analog scales; GI, gastrointestinal; KDQOL-SF, Kidney Disease and Quality of Life - Short Form; kg, kilograms; LT, liver transplantation; mg, milligram; N/A, not applicable; PHCF, partial hepactomy and cyst fenestration; PLD-Q, polycystic liver disease questionnaire; SF-36, short-form 36; wk, weeks.

### Symptom Severity Questionnaires

Six studies used a gastrointestinal (GI) symptom questionnaire, which was originally designed to capture early side effects associated with oral iron supplementation,^9^,^11^–^14^,^19^ but was not validated in patients with chronic liver disease or polycystic liver disease. The questionnaire assesses the severity of 11 symptoms on a 7-point scale, to measure abdominal pain a visual analog score (VAS) is used. It includes questions such as abdominal and epigastric pain, regurgitation, heartburn and loss of appetite. Other studies used validated disease-specific symptom severity questionnaires: polycystic liver disease questionnaire (PLD-Q) and polycystic liver disease complaint-specific assessment (POLCA).^4^,^23^ The PLD-Q is composed of 16 PLD-related symptoms (total score 0-100 points), uses a recall period of 4 weeks and was validated in Dutch and US PLD patients. Considerable overlap is observed with the POLCA which consists of 12 questions and was developed to identify PLD patients requiring liver transplantation. The POLCA questionnaire recall period for symptoms varies between present day to the last 6 months and the reproducibility of the POLCA questionnaire has not been studied.

Both PLD-Q and POLCA can be used to provide insight into PLD-related symptoms and to monitor symptom severity before and after treatment. However, it is important to note that these questionnaires are not developed to measure treatment side effects, complications, or overall HRQoL. Furthermore, both questionnaires lack a treatment threshold that can be used in clinical practice.

### HRQoL Questionnaires

The EQ-VAS is a frequently used tool in healthcare to obtain a quick global estimation of the patient’s self-rated overall HRQoL. The EQ-VAS is a visual analog scale and allows patients to rate their general health from 0 to 100. The EQ-VAS is fast and can be applied without context and the construct validity is satisfactory. However, the test-retest reliability and responsiveness of EQ-VAS is limited, and it does not provide qualitative information on symptoms the patient experiences. Using the EQ-5D a more comprehensive insight can be obtained, this questionnaire includes 5 health levels (mobility, self-care, daily activities, pain/discomfort, and anxiety/depression) and includes the EQ-VAS.^11^,^24^

For a more detailed assessment of HRQoL, the SF-36 is most frequently used in PLD research. The instrument uses a recall period of 4 weeks and can distinguish 8 different domains of HRQoL: physical functioning, social functioning, role limitations due to physical or emotional problems, mental health, energy, pain, and general health. These 8 domains can be combined into two endpoints: a PCS and a MCS score. The SF-12 is a shortened version of the SF-36 and only yields results for the summarizing PCS and MCS scores.

The European Organization for Research and Treatment of Cancer Quality of Life Questionnaire Core-30 (EORTC QLQ-C30) is a more elaborate questionnaire developed to assess general disease symptoms and HRQoL in cancer patients and has been used in PLD.^25^ Although multiple domains of HRQoL are covered, only some of the symptom-related questions pertain to PLD-specific symptoms.

Lastly, in previous studies for PLD patients the Eastern Cooperative Oncology Group performance status (ECOG-PS) has been used for PLD patients.^5^ This is a scale ranging from 0 to 5, correlating with the Karnofsky scale, that measures how well a person functions in daily life and performs activities while living with cancer. The ECOG-PS scoring is determined by the clinician or researcher and is therefore essentially different from the above discussed PROMs.

Table 2 provides an overview of the used PROMs per treatment and whether significant statistical improvement was achieved at endpoint compared with baseline. A breakdown overview of the SF-36 subdomains can be found as supplementary file 2, Supplemental Digital Content 1, http://links.lww.com/JCG/A886.

**TABLE 2:**
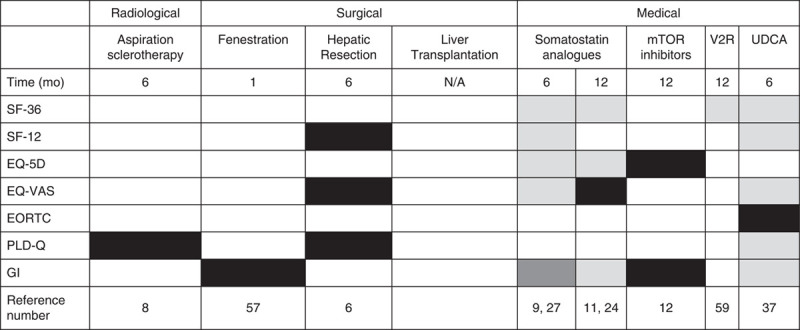
Overview of (non)Significant Changes in Patient-reported Outcome Measures in Patients With Polycystic Liver Disease Undergoing Various Treatment Options Compared With Baseline

Black = Statistical difference, Dark grey = conflicting findings, Light grey = no statistical, difference, White = no data available.

GI indicates gastrointestinal questionnaire; mTOR, mammalian target of rapamycin; UDCA, ursodeoxycholic acid etc; V2R, vasopressin-2-receptor.

## TREATMENT OPTIONS

### Indication for Treatment

Most patients with PLD are asymptomatic and the disease is frequently discovered as an incidental finding on radiologic imaging for other purposes. In PLD patients with no or only mild symptoms a conservative approach is appropriate. Female patients should be discouraged from taking exogenous estrogens as estrogen exposure is associated with liver growth. Routine surveillance is not recommended in PLD patients unless aggravation of symptoms occur.^26^

**FIGURE 1:**
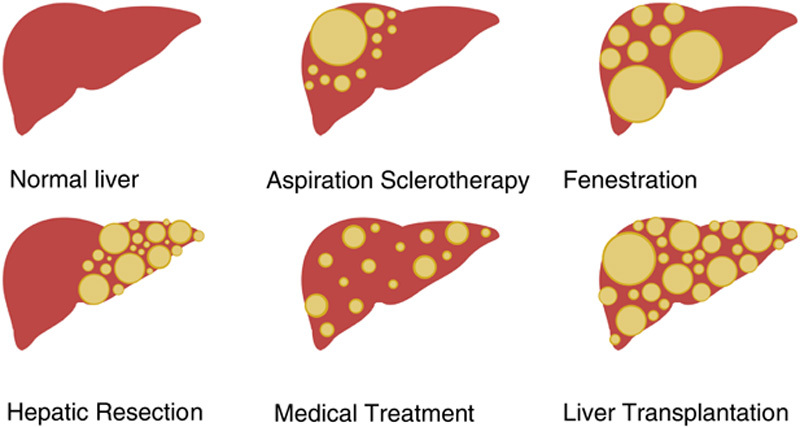
Schematic overview of the treatment options for polycystic liver disease determined on liver phenotype.

Multiple treatment therapies have been developed for symptomatic PLD patients with a decreased HRQoL. Treatment choice depends on symptoms, liver phenotype, and patient or center preferences.^26^ The different treatment options for symptomatic patients can be divided into 3 categories: radiologic therapy, surgical therapy, and medical therapy (Fig. 1).

### Radiologic Treatment

#### Aspiration Sclerotherapy

Aspiration sclerotherapy (AS) is recommended in PLD patients with a dominant or strategically located symptomatic cyst. After the cyst fluid is aspirated, a sclerosing agent is infused that aims to destroy the inner epithelial lining to prevent cyst recurrence. Median volume reduction of AS ranges between 76% and 100%^27^ and complications of AS reported are generally mild.^18^,^27^

At present no studies have been identified that investigate the effect of AS on HRQoL. The effect of this procedure on symptom severity has been discussed in a systematic review. Symptomatic relief was achieved in 72% of PLD patients, which is remarkably lower compared with the patients with solitary cysts 94%.^17^,^27^ A more recent study, not included in this systematic review, used the PLD-Q to determine symptom severity after AS in 32 PLD patients. Six months after treatment a similar proportion of patients (72%) showed an improvement in symptom scores. Patients with larger liver cyst volumes at baseline showed the best response. This study also demonstrates that improvement in PLD-Q scores determines treatment success in AS and not volume reduction.^8^

Together these studies demonstrate that AS effectively reduces symptom severity, with only mild postprocedural complications. The assumption that symptom reduction after AS also results in HRQoL improvement cannot be substantiated sufficiently at present with the available literature.

### Surgical Treatment

#### Fenestration

Cyst fenestration can be performed in patients who experience symptoms from multiple large cysts. Cyst fenestration is usually performed with a laparoscopic approach and combines internal aspiration with laparoscopic deroofing of one or more liver cysts.^28^ Fenestration is often combined with hepatic resection and little is described about mere cyst fenestration as treatment for PLD.

A recent literature review included 1314 patients, who underwent fenestration for symptomatic hepatic cysts. A subgroup analysis showed that postoperative complications, including ascites, arterial or venous bleeding, and biliary leakage, were more frequent in PLD patients (10.8% vs. 29.3%).^29^ In this review no studies were identified which used PROMs to assess the effect of cyst fenestration on HRQoL.

However, 1 study used the gastrointestinal questionnaire to assess symptoms 4 weeks before and after cyst fenestration in 12 PLD patients. Reporting of all symptoms decreased, but only the decrease of abdominal distension (*P*=0.01) and postprandial fullness (*P*=0.02) was significant. Furthermore, a correlation between liver volume and pain was found.^19^

In conclusion, only 1 small study shows that cyst fenestration has a TLV lowering property associated with decrease in pain and reduction of gastrointestinal symptoms. Currently, improvement of HRQoL in PLD patients has not been sufficiently studied.

#### Hepatic Resection

Segmental hepatic resection can be used in patients who experience severe PLD-related symptoms^6^ and have numerous cysts in several liver segments while other segments remain relatively unaffected. Resection of a polycystic liver is often combined with fenestration of the less affected segments and presents itself with technical difficulties owing to the large liver volume, altered anatomic configuration and compression of biliary or vascular structures. Owing to these challenges, high morbidity (21-51%) and mortality rates (3%) are associated.^20^,^29^–^31^

Although most articles about hepatic resection in PLD report high numbers of symptom relief (86%), these numbers are based almost exclusively on cohort studies without PROMS and merely clinical follow-up data. Only 2 studies used validated questionnaires to assess symptoms and performance status before and after surgery.

The first is a retrospective study that analyzed 29 patients who underwent partial hepatectomy combined with extensive cyst fenestration (>30). Patients’ HRQoL were classified using the ECOG-PS. Most patients (84%) were classified as category 1 (unable to perform physically strenuous activities, but ambulatory and able to complete work of a light or sedentary nature) at baseline. A postoperative liver volume reduction of 52.8% was achieved and after a mean follow-up of 70.8 ± 65 months 84% of the patients were completely asymptomatic. Overall morbidity (Clavien-Dindo grade ≥ II) rate was 41.4% and mortality was reported in 4 patients (13.8%). ECOG-PS normalized or improved in 21 patients (84%), stabilized in 3 patients (12%), and worsened in 1 patient (4%).^20^ However, as mentioned before the ECOG-PS is not a PROM but a performance status registered by the clinician.

Another prospective study included 17 PLD patients suffering from severe PLD with a median liver volume of 4781 mL (IQR: 3303–6228).^6^ Patients were subject to a combined partial hepatectomy and cyst fenestration (PHCF) for volume-related symptoms. In this study, symptom severity and HRQoL were assessed using the PLD-Q, EQ-VAS, and SF-12. Six months after surgery the median liver volume reduction was 57%. No procedure-related mortality was reported and Clavien-Dindo grade 4 complications occurred in 2 patients. An improvement in median PLD-Q score from 76.9 points to 34.8 points was reported in 13 patients. HRQoL assessed by SF-12 significantly improved with a median change in PCS score from 24.9 points to 45.7 points and a median MCS score increase from 40.5 points to 55.4 points. Clinically relevant improvement was not achieved in 3 patients (23%) after surgery. Factors that increased the risk of non-response after PHCF were low symptom burden before surgery (PLD-Q: 40.3 vs. 79.2) and with a high pre-surgical ASA classification.^6^

Although hepatic resection seems an effective volume-reducing therapy that improves HRQoL, most studies are conducted with small patient numbers and are conducted in expertise centers which precludes extrapolability to other centers. Despite the cost of a high morbidity and mortality associated with PHCF, patients with a severe symptom burden and suitable liver phenotype could be treated with this procedure.

#### Liver Transplantation

PLD is a rare indication for liver transplantation (LT) as liver function remains preserved even in the most severe cases. LT is reserved for patients with severe symptoms leading to limitations in daily life or untreatable complications, eg, severe malnutrition or portal hypertension with ascites.^5^,^28^,^32^ LT is performed as a last treatment option owing to the associated morbidity (36.5-57%), mortality (5% <30 d) and the shortage of donor organs. In ADPKD patients with a severely impaired renal function, a combined liver-kidney transplantation (CKLT) can be considered.^32^

An observational study, including 21 LT and 15 CKLT patients, studied HRQoL using the SF-36 and self-designed disease-specific questionnaires. Within the first 2 months 5 patients died. Of the remaining 28 patients (78%) who answered 91% felt better and 9% felt worse. Symptoms such as loss of appetite and vomiting, most likely resulting from enlarged liver and kidneys, significantly improved. After LT patients, SF-36 scores were not different compared with the general population.^21^ In a more recent study from China in which 11 PLD patients underwent LT, similar HRQoL outcomes were observed (PCS 87.1±6.9 and MCS 81.5±6.4).^22^

Despite considerable periprocedural morbidity and mortality, LT appears to greatly improve HRQoL in selected PLD patients. However, these findings need to be corroborated in larger cohorts.

### Medical Treatment

**FIGURE 2:**
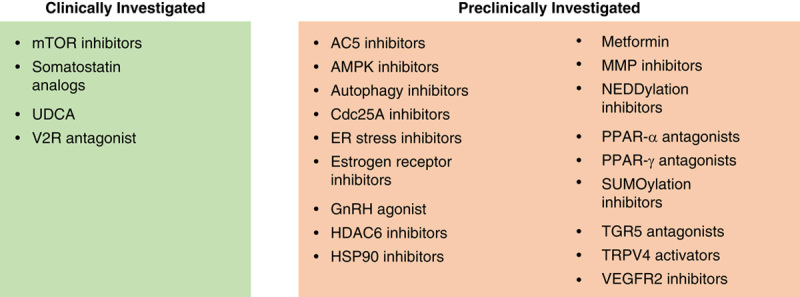
Overview of clinical and preclinical investigated therapies. AC5 indicates adenylyl cyclase 5; AMPK, adenosine monophosphate-activated protein kinase; Cdc25A, cell division cycle 25 homolog A; ER, endoplasmic reticulum; GnRH, gonadotropin hormone-releasing hormone; HDAC6, histone deacetylase 6; HSP90, heat shock protein 90; MMP, matrix metalloproteinase; mTOR, mammalian target of rapamycin; PPAR, peroxisome proliferator-activated receptor; TGR5, Takeda G protein-coupled receptor 5; TRPV4, transient receptor potential cation channel subfamily V member 4; UDCA, ursodeoxycholic acid; VEGFR2, vascular endothelial growth factor receptor 2; V2R, vasopressin 2 receptor.

The pathophysiology underlying cyst formation in ADPKD and ADPLD has not been fully elucidated. Various intracellular pathways are involved, leading to increased fluid secretion and cell proliferation in cholangiocytes and subsequently hepatic cyst formation. These pathways serve as a target for medical interventions. An overview of the (pre)clinical tested medical intervention is given in Figure 2. A concise description of the suspected pathways leading to hepatic cystogenesis in ADPKD and ADPLD are described elsewhere.^33^–^37^

#### Somatostatin Analogs

Somatostatin analogs (SAs) are the only medical treatment for PLD patients that alter the natural progression of the disease. The SAs octreotide, lanreotide and pasireotide halt hepatic cystogenesis through binding to the somatostatin receptor in cholangiocytes. Several randomized controlled trials proved that SAs effectively reduce TLV in PLD.^38^

A recent meta-analysis studied the effect of SAs on HRQoL with the SF-36 questionnaire. No significant improvement was observed in the patients receiving SAs. Only the SF-36 PCS showed a marginal, nonsignificant improvement in favor of SA (*P*=0.06).^39^ This observation is inconsistent with the hypothesis that SAs ameliorate HRQoL and symptom burden by reducing TLV. The disappointing result may be explained by the side effects that accompany SA treatment, especially in patients administered with pasireotide. After exclusion of the pasireotide trial, a pooled analysis of lanreotide and octreotide trials showed a significant improvement in the PCS compared with the control group (3.41 vs. −0.71 points, *P*=0.044), but not in the MCS (*P*=0.253).^40^ However, a subgroup analysis demonstrated detrimental side effects of octreotide and lanreotide on HRQoL. Patients with side effects had lower PCS scores (−1.32 points), while patients devoid of side effects had better scores (+4.60 points).^40^ The exact impact of SAs is difficult to assess in this study, because no symptom specific questionnaire was used.

To quantify the effect of SA treatment on symptom severity, no specific questionnaire for PLD-related symptoms has been used yet. Some studies did assess the effect of lanreotide on symptom severity using the GI questionnaire, but this is not a validated PROM in PLD patients and may not be sensitive enough to detect changes in PLD-specific symptoms. In these studies, the patient groups treated with lanreotide did not show any significant changes.^10^,^12^ Another study also used this GI questionnaire and demonstrated that lanreotide therapy significantly reduced severity of early satiety, abdominal distention, and dyspnea.^11^ However, this questionnaire is also not validated in PLD patients and therefore the precise effect of SAs on symptom severity remains hard to assess.

#### mTOR Inhibitors

Another clinically investigated medical therapy is the inhibition of the mammalian target of rapamycin (mTOR). Effects of this type of drug were reported in 2 important publications: a cohort study and a randomized controlled trial.^14^,^41^ The cohort study, consisting of kidney transplantation patients, showed an improvement in TLV, but did not assess HRQoL or symptom severity. However, kidney function and liver transplantation would have been important confounders if PROMs were administered.

Another study compared everolimus and octreotide combination therapy with octreotide monotherapy, yet showed no difference in effect on TLV.^14^ HRQoL was assessed using the GI questionnaire, the EQ-5D and EQ-VAS. No significant differences between treatment groups were found in one of the questionnaires. Interestingly, patients on octreotide monotherapy had a significant increase of EQ-VAS score relative to baseline, whereas patients in the combination therapy did not.^14^ This could be explained by the small therapeutic window and many side effects, including increased risk of infection and malignancies associated with this drug.^42^ Owing to the disappointing results from this last trial, mTOR inhibitors are not seen as an effective treatment for PLD.

#### Other Medical Treatment Options

Ursodeoxycholic acid (UDCA) is a Ca^2+^ agonist in hepatocytes and cholangiocytes and could restore intracellular Ca^2+^ homeostasis and halt hepatic cystogenesis. In a randomized controlled trial, UDCA proved ineffective in reducing TLV in PLD.^16^ The impact of UDCA on HRQoL was studied as well, using the following questionnaires: PLD-Q, SF-36, EORTC QLQ-c30, and EQ-VAS. Only the EORTC QLQ-c30 questionnaire showed a small improvement in the HRQoL, but the other questionnaires did not.^16^ Patients on UDCA therapy reported several side effects, most notably frequent stools/diarrhea, but the impact of these side effects was not evaluated. The limited effect in combination with side effects precludes application of UDCA in clinical settings.

Vasopressin-2-receptor (V2R) is involved in Ca2+-signaling pathway and inhibition of this receptor could theoretically halt hepatic cystogenesis. In a recent study the effect of tolvaptan in PLD patients was studied, but the external validity of this study is limited owing to nonrandomized retrospective design of the study. The effect of tolvaptan on TLV growth rate was not different from the control group.^43^ The effect of tolvaptan on symptom severity and HRQoL were not evaluated. Data from an ADPKD trial indicate that tolvaptan does not significantly affect HRQoL in patients who tolerate treatment beyond the first 3 months of therapy.^15^ Future randomized controlled trials are needed to provide robust evidence of the effects of V2R inhibition on TLV, HRQoL, and symptom severity in PLD. However, the effect of V2R inhibitors on PLD patients’ HRQoL is presumably limited owing to the high incidence of invasive aquaresis-related side effects such as polyuria and thirst.^44^,^45^

## FUTURE DIRECTIONS

A minority of PLD patients suffer from symptoms resulting from massive hepatomegaly. However, these symptoms may seriously impact HRQoL. In this review we discussed the effects of different treatment modalities on HRQoL and PLD-related symptoms. AS, cyst fenestration, and PHCF effectively reduce pain and symptom severity. A substantiated opinion about their impact on HRQoL cannot be provided, given the low-level of available evidence and the lack of consistency when PROMs were applied in studies. Despite limited availability of robust evidence, LT shows a significant improvement on HRQoL with the SF-36 questionnaire. The effects of medical treatments on HRQoL are not yet fully elucidated. A beneficial effect on HRQoL was found in some studies, while others demonstrate a negative effect of treatment-associated side effects. The volume-reducing effects of medical treatment can also be too modest or too slow compared with surgical therapies to detect tangible changes in HRQol.

The effects of PLD treatment are expected to be similar to other diseases defined by mass-related effects (eg, liver adenomas, gastrointestinal neuroendocrine tumors, and giant hemangiomatosis). Unfortunately, no evidence is available that discusses HRQoL or symptom severity in liver adenomas or gastrointestinal neuroendocrine tumors. Two studies demonstrated a positive effect of surgical treatment on HRQoL scores in giant hemangioma patients,^46^,^47^ comparable to surgical treatment in PLD.

Most studies discussed in this review used TLV or cyst volume reduction as their primary outcome, while the end goal of treatment is to reduce symptom burden. Development and validation of PLD-specific PROMs allowed the use of these instruments as appropriate primary outcome measures to determine treatment impact in PLD. Despite this progress, there are still a number of issues that need to be addressed before use of PROMs as a bonafide primary outcome (1) general HRQoL versus symptom specific questionnaires, (2) differences in application of questionnaires, and (3) determination of PROM thresholds for treatment guidance

Throughout this review we have made an important distinction between PROMs aimed at HRQoL and PROMs aimed at symptom severity. HRQoL questionnaires measure the physical, mental and social functioning related to an individual’s health. Symptom severity questionnaires address the disease-specific symptoms which could affect HRQoL. PLD therapies should be aimed at reduction of PLD-specific symptoms, thereby ideally also improving HRQol. However, one should keep in mind that symptom severity questionnaires are not validated to measure treatment-associated side effects, which might underestimate a potential negative impact of PLD therapies on overall HRQoL. Currently, no questionnaire is available that can measure both HRQoL and symptom severity in PLD patients. For this reason, we recommend using a combination of both types of questionnaires. As discussed in this review, several validated questionnaires are appropriate to determine symptoms severity and HRQoL, each of which has its (dis)advantages and applicability depends on your research question.

Another difficulty in assessing the effect of PLD treatment using PROMS are the differences in how questionnaires are applied. Different questionnaires and different lengths of follow-up periods are used to measure symptoms or HrQol in the studies that address this issue which complicates a comparison of therapy outcomes. We advise baseline measurements before each treatment. The administration frequency of a PROM during medical treatment or after radiologic and surgical treatment should be done in a standardized manner, considering associated side effects, and the length of treatment and individual treatment outcome measures. By frequently administering the questionnaires, recurrence of symptoms can be detected. Treatment specific side effects and complications should also be monitored during checkups. Table 3 can be used to determine whether one or multiple selected questionnaires can sufficiently aid in research to determine (change in) HRQoL related to PLD treatment dependent of your goals.

**TABLE 3:** Suggested Features for PROMS Assessing Symptoms, Side Effects, and HRQoL Related to PLD Treatment

Feature	Need to Have	Nice to Have
Validation	Validated	—
Symptoms	Assesses PLD-specific symptoms (yes/no)	Includes threshold to select patients for treatment
HRQoL	Provides an overall indication of HRQoL Can detect clinically relevant changes in HRQoL	Includes specific HRQoL subdomains (ie, emotional health, health perception)
Complications and side effects	Includes most frequent complications and side-effects associated with the treatment	Includes options to report unexpected side effects or complications
Time needed	≤30 minutes	≤15 minutes
Language	Language use understandable for the entire population	Available in multiple languages to enable cross-country comparability
Frequency of administration (medical therapy)	At baseline and after stopping the treatment	During treatment with an appropriate interval depending on the therapy During follow-up period after stop treatment Treatment-specific effects on symptom burden, HRQoL and complications should be taken into account when administering the PROM during follow-up
Frequency of administration (radiologic and surgical)	Baseline and at the end of follow-up	Treatment-specific effects on symptom burden, HRQoL and complications should be taken into account when administering the PROM during follow-up

Previous studies discussed that treatment initiation is based on symptoms and not liver phenotype. This opens up the possibility to use PROMs in the clinical setting to determine which patients should start or discontinue a certain treatment. To minimize variation between centers and physicians, PROM thresholds for therapies should be determined. Patients with a score exceeding this threshold would be eligible for treatment or inclusion in studies. Liver phenotype can aid in the determination of treatment type. Adequate selection of patients can maximize treatment efficacy and reduce the frequency of unwanted and needless complications. Although at present questionnaires have no thresholds to determine treatment initiation, our recommendation is to administer PROMs when monitoring symptomatic PLD. This provides insight in the patient’s natural disease progression and allows for an objective method to capture dynamics in symptom presentation.

In conclusion, validated questionnaires can aid physicians when integrated in daily practice in a uniform and consistent approach. The next step is to determine clinically relevant PROM thresholds. Especially disease-specific PROMS could assist physicians to (1) determine when treatment is indicated, (2) evaluate treatment efficacy more objectively, and (3) swiftly identify symptom recurrence after treatment in PLD patients.

## Supplementary Material

SUPPLEMENTARY MATERIAL

Supplemental Digital Content is available for this article. Direct URL citations appear in the printed text and are provided in the HTML and PDF versions of this article on the journal's website, www.jcge.com.

## References

[1] Van Keimpema L De Koning DB Van Hoek B . Patients with isolated polycystic liver disease referred to liver centres: clinical characterization of 137 cases. Liver Int. 2011;31:92–98. 20408955 10.1111/j.1478-3231.2010.02247.x

[2] Abu-Wasel B Walsh C Keough V . Pathophysiology, epidemiology, classification and treatment options for polycystic liver diseases. World J Gastroenterol. 2013;19:5775–5786. 24124322 10.3748/wjg.v19.i35.5775 PMC3793132

[3] Neijenhuis MK Kievit W Verheesen SM . Impact of liver volume on polycystic liver disease-related symptoms and quality of life. United Eur Gastroenterol J. 2018;6:81–88. 10.1177/2050640617705577 PMC5802666 29435317

[4] Neijenhuis MK Gevers TJ Hogan MC . Development and validation of a disease-specific questionnaire to assess patient-reported symptoms in polycystic liver disease. Hepatology. 2016;64:151–160. 26970415 10.1002/hep.28545 PMC4917464

[5] Schnelldorfer T Torres VE Zakaria S . Polycystic liver disease: a critical appraisal of hepatic resection, cyst fenestration, and liver transplantation. Ann Surg. 2009;250:112–118. 19561475 10.1097/SLA.0b013e3181ad83dc

[6] Bernts LHP Neijenhuis MK Edwards ME . Symptom relief and quality of life after combined partial hepatectomy and cyst fenestration in highly symptomatic polycystic liver disease. Surgery. 2020;168:25–32. 32402542 10.1016/j.surg.2020.02.014 PMC7347464

[7] Wijnands TF Neijenhuis MK Kievit W . Evaluating health-related quality of life in patients with polycystic liver disease and determining the impact of symptoms and liver volume. Liver Int. 2014;34:1578–1583. 24313956 10.1111/liv.12430

[8] Neijenhuis MK Wijnands TFM Kievit W . Symptom relief and not cyst reduction determines treatment success in aspiration sclerotherapy of hepatic cysts. Eur Radiol. 2019;29:3062–3068. 30542749 10.1007/s00330-018-5851-y PMC6510865

[9] van Keimpema L Nevens F Vanslembrouck R . Lanreotide reduces the volume of polycystic liver: a randomized, double-blind, placebo-controlled trial. Gastroenterology. 2009;137:1661–1668; e1661–1662. 19646443 10.1053/j.gastro.2009.07.052

[10] Hogan MC Masyuk TV Page LJ . Randomized clinical trial of long-acting somatostatin for autosomal dominant polycystic kidney and liver disease. J Am Soc Nephrol. 2010;21:1052–1061. 20431041 10.1681/ASN.2009121291 PMC2900957

[11] Gevers TJ Hol JC Monshouwer R . Effect of lanreotide on polycystic liver and kidneys in autosomal dominant polycystic kidney disease: an observational trial. Liver Int. 2015;35:1607–1614. 25369108 10.1111/liv.12726

[12] van Aerts RMM Kievit W D’Agnolo HMA . Lanreotide reduces liver growth in patients with autosomal dominant polycystic liver and kidney disease. Gastroenterology. 2019;157:481–491.e487. 31022403 10.1053/j.gastro.2019.04.018

[13] Hogan MC Chamberlin JA Vaughan LE . Pansomatostatin agonist pasireotide long-acting release for patients with autosomal dominant polycystic kidney or liver disease with severe liver involvement: A Randomized Clinical Trial. Clin J Am Soc Nephrol. 2020;15:1267–1278. 32843370 10.2215/CJN.13661119 PMC7480539

[14] Chrispijn M Gevers TJ Hol JC . Everolimus does not further reduce polycystic liver volume when added to long acting octreotide: results from a randomized controlled trial. J Hepatol. 2013;59:153–159. 23499726 10.1016/j.jhep.2013.03.004

[15] Anderegg MA Dhayat NA Sommer G . Quality of life in autosomal dominant polycystic kidney disease patients treated with tolvaptan. Kidney Med. 2020;2:162–171. 32964204 10.1016/j.xkme.2019.11.008 PMC7487949

[16] D’Agnolo HM Kievit W Takkenberg RB . Ursodeoxycholic acid in advanced polycystic liver disease: a phase 2 multicenter randomized controlled trial. J Hepatol. 2016;65:601–607. 27212247 10.1016/j.jhep.2016.05.009

[17] Benzimra J Ronot M Fuks D . Hepatic cysts treated with percutaneous ethanol sclerotherapy: time to extend the indications to haemorrhagic cysts and polycystic liver disease. Eur Radiol. 2014;24:1030–1038. 24563160 10.1007/s00330-014-3117-x

[18] Ogawa K Fukunaga K Takeuchi T . Current treatment status of polycystic liver disease in Japan. Hepatol Res. 2014;44:1110–1118. 24308726 10.1111/hepr.12286

[19] van Keimpema L Ruurda JP Ernst MF . Laparoscopic fenestration of liver cysts in polycystic liver disease results in a median volume reduction of 12.5%. J Gastrointest Surg. 2008;12:477–482. 17957434 10.1007/s11605-007-0376-8

[20] Boillot O Cayot B Guillaud O . Partial major hepatectomy with cyst fenestration for polycystic liver disease: Indications, short and long-term outcomes. Clin Res Hepatol Gastroenterol. 2021;45:101670. 33722781 10.1016/j.clinre.2021.101670

[21] Kirchner GI Rifai K Cantz T . Outcome and quality of life in patients with polycystic liver disease after liver or combined liver-kidney transplantation. Liver Transpl. 2006;12:1268–1277. 16741930 10.1002/lt.20780

[22] Ding F Tang H Zhao H . Long-term results of liver transplantation for polycystic liver disease: single-center experience in China. Exp Ther Med. 2019;17:4183–4189. 31007749 10.3892/etm.2019.7449 PMC6468458

[23] Temmerman F Dobbels F Ho TA . Development and validation of a polycystic liver disease complaint-specific assessment (POLCA). J Hepatol. 2014;61:1143–1150. 24996047 10.1016/j.jhep.2014.06.024

[24] Natale P Hannan E Sautenet B . Patient-reported outcome measures for pain in autosomal dominant polycystic kidney disease: a systematic review. PLoS One. 2021;16:e0252479. 34043715 10.1371/journal.pone.0252479 PMC8158964

[25] Kamphues C Rather M Engel S . Laparoscopic fenestration of non-parasitic liver cysts and health-related quality of life assessment. Updates Surg. 2011;63:243–247. 21927951 10.1007/s13304-011-0110-7

[26] van Aerts RMM van de Laarschot LFM Banales JM . Clinical management of polycystic liver disease. J Hepatol. 2018;68:827–837. 29175241 10.1016/j.jhep.2017.11.024

[27] Wijnands TF Görtjes AP Gevers TJ . Efficacy and safety of aspiration sclerotherapy of simple hepatic cysts: a systematic review. Am J Roentgenol. 2017;208:201–207. 27824501 10.2214/AJR.16.16130

[28] Drenth JP Chrispijn M Nagorney DM . Medical and surgical treatment options for polycystic liver disease. Hepatology. 2010;52:2223–2230. 21105111 10.1002/hep.24036

[29] Bernts LHP Echternach SG Kievit W . Clinical response after laparoscopic fenestration of symptomatic hepatic cysts: a systematic review and meta-analysis. Surg Endosc. 2019;33:691–704. 30334152 10.1007/s00464-018-6490-8 PMC6394680

[30] Schindl MJ Redhead DN Fearon KC . The value of residual liver volume as a predictor of hepatic dysfunction and infection after major liver resection. Gut. 2005;54:289–296. 15647196 10.1136/gut.2004.046524 PMC1774834

[31] Chebib FT Harmon A Irazabal Mira MV . Outcomes and durability of hepatic reduction after combined partial hepatectomy and cyst fenestration for massive polycystic liver disease. J Am Coll Surg. 2016;223:118–126.e111. 27016902 10.1016/j.jamcollsurg.2015.12.051 PMC4967356

[32] Lerut J Ciccarelli O Rutgers M . Liver transplantation with preservation of the inferior vena cava in case of symptomatic adult polycystic disease. Transpl Int. 2005;18:513–518. 15819798 10.1111/j.1432-2277.2005.00061.x

[33] Perugorria MJ Masyuk TV Marin JJ . Polycystic liver diseases: advanced insights into the molecular mechanisms. Nat Rev Gastroenterol Hepatol. 2014;11:750–761. 25266109 10.1038/nrgastro.2014.155 PMC4526263

[34] Wills ES Roepman R Drenth JP . Polycystic liver disease: ductal plate malformation and the primary cilium. Trends Mol Med. 2014;20:261–270. 24506938 10.1016/j.molmed.2014.01.003

[35] Masyuk TV Masyuk AI LaRusso NF . Polycystic liver disease: advances in understanding and treatment. Annu Rev Pathol. 2022;17:251–269. 34724412 10.1146/annurev-pathol-042320-121247 PMC8842879

[36] Lee-Law PY Olaizola P Caballero-Camino FJ . Inhibition of NAE-dependent protein hyper-NEDDylation in cystic cholangiocytes halts cystogenesis in experimental models of polycystic liver disease. United Eur Gastroenterol J. 2021;9:848–859. 10.1002/ueg2.12126 PMC8435261 34310849

[37] Lee-Law PY Olaizola P Caballero-Camino FJ . Targeting UBC9-mediated protein hyper-SUMOylation in cystic cholangiocytes halts polycystic liver disease in experimental models. J Hepatol. 2021;74:394–406. 32950589 10.1016/j.jhep.2020.09.010 PMC8157180

[38] Suwabe T Barrera FJ Rodriguez-Gutierrez R . Somatostatin analog therapy effectiveness on the progression of polycystic kidney and liver disease: a systematic review and meta-analysis of randomized clinical trials. PLoS One. 2021;16:e0257606. 34559824 10.1371/journal.pone.0257606 PMC8462725

[39] Garofalo C Capuano I Pennino L . The effects of somatostatin analogues on liver volume and quality of life in polycystic liver disease: a meta-analysis of randomized controlled trials. Sci Rep. 2021;11:23500. 34873228 10.1038/s41598-021-02812-z PMC8648823

[40] Neijenhuis MK Gevers TJ Nevens F . Somatostatin analogues improve health-related quality of life in polycystic liver disease: a pooled analysis of two randomised, placebo-controlled trials. Aliment Pharmacol Ther. 2015;42:591–598. 26129925 10.1111/apt.13301

[41] Qian Q Du H King BF . Sirolimus reduces polycystic liver volume in ADPKD patients. J Am Soc Nephrol. 2008;19:631–638. 18199797 10.1681/ASN.2007050626 PMC2391057

[42] Pallet N Legendre C . Adverse events associated with mTOR inhibitors. Expert Opin Drug Saf. 2013;12:177–186. 23252795 10.1517/14740338.2013.752814

[43] Mizuno H Sekine A Suwabe T . Potential effect of tolvaptan on polycystic liver disease for patients with ADPKD meeting the Japanese criteria of tolvaptan use. PLoS One. 2022;17:e0264065. 35176098 10.1371/journal.pone.0264065 PMC8853523

[44] Torres VE Chapman AB Devuyst O . Tolvaptan in later-stage autosomal dominant polycystic kidney disease. N Engl J Med. 2017;377:1930–1942. 29105594 10.1056/NEJMoa1710030

[45] Torres VE Chapman AB Devuyst O . Tolvaptan in patients with autosomal dominant polycystic kidney disease. N Engl J Med. 2012;367:2407–2418. 23121377 10.1056/NEJMoa1205511 PMC3760207

[46] Qiu J Chen S Wu H . Quality of life can be improved by surgical management of giant hepatic haemangioma with enucleation as the preferred option. HPB (Oxford). 2015;17:490–494. 25728743 10.1111/hpb.12391 PMC4430778

[47] Liu Q Liu F Ding J . Surgical outcomes and quality of life between laparoscopic and open approach for hepatic hemangioma: a propensity score matching analysis. Medicine (Baltimore). 2019;98:e14485. 30732219 10.1097/MD.0000000000014485 PMC6380717

