# Supplementary material for: Treatment of Polycystic Liver Disease: Impact on Patient-reported Symptom Severity and Health-related Quality of Life
Source: J Clin Gastroenterol. 2022 Aug 19;56(9):731–9. doi: 10.1097/MCG.0000000000001749 (PMC9432811; doi:10.1097/MCG.0000000000001749)
Supplement: SUPPLEMENTARY MATERIAL [file mcg-56-731-s001.docx]

**Supplementary File 1**

PubMed was searched using the following search terms:'Polycystic liver disease' AND 'Quality of life' OR: 'Somatostatin analogues', ‘Lanreotide’, ‘Octreotide’, ‘Pasireotide’, ‘mTOR inhibitors’, ‘UDCA’, ‘V2R antagonist’, ‘Tolvaptan’, ‘Aspiration’, ‘Aspiration sclerotherapy’,’Fenestration’, ‘Cyst fenestration’, ‘Hepatic resection’, ‘PHCF’, ‘Liver transplantation’, ‘LTx’.

**Supplementary File 2**

| SF-36 subdomains | Medical | | | |
| --- | --- | --- | --- | --- |
|  | Somatostatin analogues | | V2R | UDCA |
| Time (mo) | 6 | 12 | 12 | 6 |
|  |  |  |  |  |
| Physical functioning |  |  |  |  |
| Social functioning |  |  |  |  |
| Physical role |  |  |  |  |
| Emotional role |  |  |  |  |
| Mental health |  |  |  |  |
| Vitality |  |  |  |  |
| Bodily pain |  |  |  |  |
| Health perception |  |  |  |  |
| General health |  |  |  |  |
| Reference number | 9, 27 | 11, 24 | 59 | 37 |

**Table: Breakdown overview of (non)significant changes in health-related quality of life measured with the Short Form 36 in patients with polycystic liver disease using somatostatin analogues, vasopressin-2-receptor or ursodeoxycholic acid compared with baseline.**

Legenda: V2R = vasopressin-2-receptor, UDCA = ursodeoxycholic acid etc., Black = Statistical difference, Light grey = no statistical, difference , White = no data available
